# Early Versus Delayed Surgery in Gallstone Pancreatitis

**DOI:** 10.1155/1989/53232

**Published:** 1989

**Authors:** William C. Dooley, John L. Cameron

**Affiliations:** Department of Surgery The Johns Hopkins Hospital Baltimore, Maryland USA


*HPB surgery* 1989. Vol. 1. pp. 363–373
Reprints available directly from the publisher
Photocopying permitted by license only

© 1989 Harwood Academic Publishers GmbH
Printed in Great Britain

### HPB INTERNATIONAL

**EDITORIAL & ABSTRACTING SERVICE**

JOHN TERBLANCHE, EDITOR
Department of Surgery,
Medical School · Observatory
Cape Town · South Africa
Telephone: Department Office: 47–1250
Hospital Connection: 47–3311
Telex: 5–22208
Tel Add: ALUMNI CAPETOWN
Telefax: (021) 47–8955

### EARLY VERSUS DELAYED SURGERY IN GALLSTONE PANCREATITIS

### ABSTRACT

*T.R. Kelly, D.S. Wagner. (1988) Gallstone pancreatitis: a prospective randomized trial of the timing of surgery. Surgery, 104, 600–605.*

**The correct timing of surgery in cases of gallstone pancreatitis is debatable. To delineate more clearly the influence of the timing of surgery in the treatment of the disease, a prospective randomized clinical study of early surgery (less than 48 hours after admission) and delayed surgery (more than 48 hours after admission) was conducted in 165 patients. Ranson's prognostic signs of severity of disease were used to classify the patients into two risk groups: mild pancreatitis (three or fewer positive signs) and severe pancreatitis (more than three positive signs). In patients with three or fewer positive Ranson's signs, the time of surgery appeared to have little effect on the outcome, whereas in patients with more than three positive signs, early surgery resulted in a significant increase in rates of morbidity and mortality. Controlled randomization showed that in patients with gallstone pancreatitis, edematous or hemorrhagic necrotizing pancreatitis can develop, with or without inpacted stones, early or late in the progression of the disease, during early or delayed surgery. These findings suggest that (1) although a gallstone initiates a bout of pancreatitis, it does not cause the progression of the disease; (2) the fate of the progression of pancreatitis is decided early by the amount of digestive enzymes being activated; (3) early removal of an impacted stone does not ameliorate the progression of pancreatitis; and (4) surgery should be performed during the initial hospital admission after the pancreatitis has subsided.**

KEYWORDS: Gallstone pancreatitis, biliary surgery, endoscopic sphincterotomy

### PAPER DISCUSSION

The timing of interventional therapies in the management of gallstone pancreatitis continues to be a much debated issue. Studies addressing the timing of surgery have been seriously hampered by small numbers of patients presenting with a disease of widely varying clinical severity. The broad spectrum of this disease has lead to a wide variety of management styles to which proponents of each attribute success. Prospective trials with large numbers of patients, such as that of Kelly and Wagner, are needed to sort through this increasingly complex range of management recommendations. They have partially addressed the crucial issue of widely varying clinical severity by stratifying patients by Ranson's criteria.

Previous studies have not been as convincing regarding recommendations for the timing of surgery, for a variety of reasons including the lack of severity stratification. Ranson presented data from a retrospective non-randomized study in the late 1970s which tended to show benefit of delayed operation in the most severe cases of gallstone pancreatitis[1]. His definition of early surgery (within one week of admission) was however fairly generous and patients were not randomized to any particular treatment protocol. Most retrospective studies have agreed with his conclusions[2-4]. They have suggested that most patients pass the gallstone and clinically improve without the need for surgery. In patients with mild disease, all studies have shown that the timing of surgery is not important. However, these retrospective studies have shown that, with sick patients, early surgery seems to be associated with high risk. Whether the high mortality and morbidity of this group was a direct result of surgery or represented a selection bias toward intervention in the sickest patients, was not clear.

In contrast to most reports, Stone's group found no adverse effects of early operation in a prospective randomized trial[5]. They randomized a group of 65 patients to operation within 73 hours of admission or to conservative management with eventual operation three months after the acute admission. Their operative procedure represented one of the most aggressive recommendations — cholecystectomy, transduodenal sphincteroplasty, and pancreatic duct septostomy. A major flaw in this study, however, is the lack of stratification by clinical severity of disease, since previous studies showed that most patients have mild disease and would get better with expectant management only. Most studies, such as that of Kelly and Wagner, place the incidence of severe cases (as defined by greater than three Ranson's criteria) to be in the range of 25%. The numbers of patients in Stone's study, 36 in the early operation group and 29 in the late group, are so small that the impact of any such aggressive intervention on the morbidity and mortality of the high-risk patients cannot be clearly or statistically evaluated with any reasonable degree of certainty. Further, some of the poor outcomes in the late operation group might be blamed on the unusually long delay past the resolution of acute symptoms before cholecystectomy. This study leaves unanswered the role of surgery in the sickest patients while further confirming the previous observation that the timing of surgery is unimportant to the vast majority of low-risk patients.

Kelly presents an elegant and larger prospective trial where patients were randomized to operation within 48 hours of admission or after 48 hours. Further, the patients were stratified for severity by Ranson's criteria. The previous suspected relationship of early surgery with increased morbidity and mortality for the patients with the most severe pancreatitis was proven. The morbidity of those patients with greater than three Ranson's criteria was 19/23, or 83%, for early operation as compared to 3/17, or 18%, for late operation. Further, the mortality statistics are even more striking with 11/23, or 48%, mortality in the early group compared to 2/17, or

11%, mortality in the late operation group. This study used the more conventional operative techniques of cholecystectomy and operative cholangiogram with common bile duct exploration and sphincteroplasty being reserved only for those patients with positive cholangiography. Although this study dissuades from early intervention in the severe cases, it must be pointed out that Stone's study did take a more aggressive tack with the inclusion of operative sphincteroplasty in all cases. It is interesting to note that the natural urge for intervention in severe cases led to the use of ERCP in eight patients and papillotomy in one of Kelly's late operation group. Although the claim is made that an obstructing gallstone does not worsen the disease and that removal of that obstructing stone does not ameliorate the progression of the disease, there is no clear-cut data from this study to prove these points. The fact that patients did worse in the early operation group may reflect only that this group of seriously ill patients can tolerate less well the insults of surgery and anesthesia and their associated profound changes in metabolism, immunity, etc. What is clear from this study is that surgical intervention without papillotomy should be delayed, and delayed probably to the point that symptoms have subsided.

ERCP and endoscopic papillotomy are other possible options in the management of gallstone pancreatitis. Safrany and Cotton suggested this therapy with a small and optimistic series in 1980[6]. Since that time, several studies have looked at endoscopic management with findings of routinely low mortality and morbidity[7–9]. Neoptolemos and associates performed a prospective randomized trial of ERCP with papillotomy versus conventional expectant mangement with stratification of patients by the Glasgow criteria[10]. They had only twenty patients who were classified as severe. In that group, 12 were managed conventionally and 8 with urgent ERCP/ES. Complications occurred in 5/12 conventional and 2/8 ERCP patients and the only deaths were two in the conventionally managed group. These results, when combined with other larger retrospective European series, show clearly that this technique can be safely used in the sickest patients[7–9, 11]. It is possible that this therapy when performed early will reduce the incidence of the most severe complications and deaths in those patients with the worst prognosis as judged by the Glasgow criteria. This cannot be said conclusively, however, without more patients and a larger trial. This method of management has become popular in Europe because of its apparent safety.

The real unanswered question is whether or not early relief of ductal obstruction in the sickest patients can change the natural history of their disease and reduce their morbidity and mortality. Experimental studies seem to indicate that once the initial injury has occurred, nothing alters the course of the disease[12]. Stone and Neoptolemos have both concluded that early relief of this ductal obstruction will change the course of the most severe forms of gallstone pancreatitis. Although not clearly proven from their data, this is a point which deserves further investigation. Since early surgical intervention is associated with high risks by most groups and endoscopic intervention associated with low risks, the optimal study would be to randomize the sickest patients ($>3$ Ranson's criteria) to early endoscopic intervention versus conventional management with delayed surgery in a large multi-center prospective trial. A study such as this might answer our lingering question concerning persistent ductal obstruction and its relationship to clinical outcome for the most severe forms of gallstone pancreatitis.

William C. Dooley, M.D.
John L. Cameron, M.D.
Department of Surgery
The Johns Hopkins Hospital
Baltimore, Maryland

### REFERENCES

1. Ranson, J.H.C. (1979) The timing of biliary tract surgery in acute pancreatitis. *Ann. Surg*; **189**: 654–663.
2. Semel, L., Schrieber, D., Fromm, D. (1983) Gallstone pancreatitis. *Archiv. Surg*; **118**: 901–904.
3. Tondelli, P., Stutz, K., Harder, F., Schuppisser, J-P., Allgower, M. (1982) Acute gallstone pancreatitis: best timing for surgery. *Br. J. Surg;* **69**, 709–710.
4. Kim, U., Shen, H-Y., Bodner, B. (1988) Timing of surgery for actue gallstone pancreatitis. *Am. J. Surg;* **156**: 393–396.
5. Stone, H.H., Fabian, T.C., Dunlop, W.E. (1981) Gallstone pancreatitis. *Ann. Surg;* **195**: 305–312.
6. Safrany, L., Cotton, P.B. (1981) A preliminary report: urgent duodenoscopic sphincterotomy for acute gallstone pancreatitis. *Surgery:* **89**: 424–428.
7. Seigal, J.H., Tone, P., Menikeim, D. (1986) Gallstone pancreatitis: pathogenesis and clinical forms — the emerging role of endoscopic management. *Am. J. Gastroenterol*; **81**: 774–778.
8. Belghiti, J., Kleinman, P., Cherqui, D., Pernceni, T., Bernades, P., Fekete, F. (1987) Traitement precoce de la lithiase biliaire au cours des pancreatites biliaires. *Gastroenterol. clin Biol*; **11**: 786–789.
9. Rosseland, A.R., Solhaug, J.H. (1984) Early or delayed endoscopic papillotomy in gallstone pancreatitis. *Ann. Surg*; **199**: 165–167.
10. Neoptolemos, J.P., London, N. Slater, N.D., Carr-Locke, D.C., Fossard, D.P., Moosa, A.R. (1986) A prospective study of ERCP and endoscopic sphincterotomy in the diagnosis and treatment of gallstone acute pancreatitis. *Archiv. Surg*; **121**: 697–702.
11. Neoptolemos, J.P., Carr-Locke, D.L., London, N., Bailey, I. Fossard, D.P. (1988) ERCP findings and the role of endoscopic sphincterotomy in acute gallstone pancreatitis. *Br. J. Surg*; **75**: 954–960.
12. Sawfey, H., Bulkley, G.B., Cameron, J.L. (1984) The role of oxygen-derived free radicals in the pathogenesis of acute pancreatitis. *Ann. Surg;* **200**: 405–413.

### LIVER TRANSPLANTATION IN THE TREATMENT OF BLEEDING OESOPHAGEAL VARICES

### ABSTRACT

*S. Iwatsuki, T.E. Starzl, S. Todo, R.D. Gordon, A.G. Tzakis, J.W. Marsh, L. Makowka, B. Koneru, A. Stieber, G. Klintmalm, B. Husberg, D. van Thiel. (1988) Liver transplantation in the treatment of bleeding esophageal varices. Surgery, 104; 697–705.*

**From March 1980 to July 1987, 1000 patients with various end-stage liver diseases received orthotopic liver transplants. Of the 1000 patients, three hundred two had definite histories of bleeding from esophageal varices before transplantation. There were 287 patients with nonalcoholic liver diseases and 15 patients with alcoholic cirrhosis. All patients had very poor liver function, which was the main indication for liver transplantation. One- through 5-year actuarial survival rates of the 302 patients were 79%, 74%, 71%, 71%, and 71%, respectively. These survival rates are far better than those obtained with other available modes of treatment for bleeding varices when liver disease is advanced. Long-term sclerotherapy is the treatment of primary choice for bleeding varices. Patients in whom sclerotherapy fails should be considered for liver transplantation unless clear contraindications exist.**

---

KEYWORDS: Liver transplantation; sclerotherapy; portal hypertension; portosystemic shunts

